# Nursing Leadership During COVID-19: Leading an Inpatient Response at a Regional Military Medical Center

**DOI:** 10.1093/milmed/usab179

**Published:** 2021-09-01

**Authors:** Ekerette U Akpan, William O Murray, Mario A Vergara, Sarah J Murray, Christopher H Stucky, Marla J De Jong, Elba Villacorta

**Affiliations:** Department of Nursing, Landstuhl Regional Medical Center, APO, AE 09180, USA; Department of Nursing, Landstuhl Regional Medical Center, APO, AE 09180, USA; Department of Nursing, Landstuhl Regional Medical Center, APO, AE 09180, USA; Virtual Health, Regional Health Command, Europe, APO, AE 09180, USA; Department of Nursing, Womack Army Medical Center, Fort Bragg, NC 28310-7301, USA; University of Utah College of Nursing, Salt Lake City, UT 84112-5880, USA; Public Health Command Pacific Region, Ft. Shafter, HI 96859, USA

## Abstract

The coronavirus disease 2019 pandemic stressed healthcare systems worldwide and exposed major flaws in military and civilian healthcare systems. Landstuhl Regional Medical Center (LRMC) serves as the only military medical center for over 205,000 U.S. service members, beneficiaries, and coalition partners stationed throughout Europe, Africa, and the Middle East. The pandemic response required LRMC leaders to reconfigure services to meet pandemic concerns while providing lifesaving care to injured service members from combatant commands. The quickly evolving pandemic challenged leaders to ensure healthcare delivery amid constant change and imperfect information. While LRMC senior leaders developed a strategic pandemic response plan, a multidisciplinary team of nurses, doctors, and technicians collaborated to create an inpatient team to support the dual mission of coronavirus disease 2019 response and casualty care for the warfighter. In this manuscript, we discuss how a multidisciplinary clinical working group at a regional medical center prepared and responded to the pandemic, strategically planned patient care, and ensured support to combatant commands for ongoing forward military operations. Additionally, we share our experiences and lessons learned to inform other military facilities across the medical community and global healthcare systems.

## INTRODUCTION

The coronavirus disease 2019 (COVID-19) first emerged in China and quickly spread to cause a worldwide pandemic, resulting in 124 million global cases and over 2.7 million deaths.^1^ The severity of the disease varies from flu-like symptoms to severe acute respiratory syndrome.^2^ The disease spreads among people in close contact through respiratory droplets or small particles, such as those in aerosols produced during coughing, sneezing, talking, or medical procedures. The European Centre for Disease Prevention and Control reported the first case of COVID-19 on January 27, 2020, near Munich, Bavaria.^3^ All European Union countries soon reported COVID-19 cases by March 25, 2020,^3^ and Germany imposed a nationwide state of emergency for the first time in its postwar history.^4^ Landstuhl Regional Medical Center (LRMC) leaders reported their first COVID-19-positive patient in March 2020, and they developed strategies to prepare for a potential rapid influx of patients.

The pandemic stressed healthcare systems worldwide and exposed major weaknesses, including severe shortages of personal protective equipment (PPE), hospital beds, medication, and supplies.^5,6^ COVID-19 also challenged the vitality of the healthcare workforce, as the disease infected thousands of U.S. healthcare workers, resulting in the mortality of over 3600 frontline clinicians.^7^ The pandemic likewise affected military healthcare, as military leaders enacted measures to ensure safe and effective care to their beneficiaries.^8^

LRMC is a forward stationed U.S. military medical center located in western Germany, where over 205,000 beneficiaries, including U.S. and coalition forces, Department of State personnel, repatriated U.S. citizens, and dependents receive care.^9^ LRMC is the largest American hospital outside the continental US. Its inpatient capability includes a combined intensive care unit (ICU), medical–surgical pediatric ward, labor and delivery ward, mother–baby unit, and neonatal ICU. LRMC has a unique mission as an evacuation and treatment center for all injured U.S. service members and civilians, including coalition forces supporting the Africa Command, Central Command, and European Command.

COVID-19 created a heightened sense of urgency, as a neighboring country, Italy, was a pandemic hotspot inundated with critically ill patients that threatened to overwhelm their healthcare system.^10^ The risk to LRMC beneficiaries was elevated due to the infectiousness of the virus, mortality rate, and potential impact of diverting efforts away from the primary mission. Furthermore, the German healthcare system did not have the capacity to assume medical care for U.S. service members and their dependents if their hospitals became overwhelmed. In this manuscript, we discuss how a multidisciplinary (Multi-D) inpatient team at a regional medical center prepared and responded to the pandemic while continuing to manage combat casualty care. Additionally, we share our experiences and lessons learned to inform other military facilities across the medical community and global healthcare systems.

## BUILDING THE TEAM

Nursing leaders from inpatient wards collaborated with infectious disease, pulmonary, and critical care medicine teams, and created an ad-hoc COVID-19 Inpatient Working Group (CIWG) to leverage all available expertise for a coordinated pandemic response. Comprising the CIWG were bedside clinical staff, clinical leaders, and ancillary staff. Key CIWG leaders and members were the Deputy Commander for Inpatient Services (DCIS) and leadership from inpatient nursing services, critical care, pulmonology medicine, women’s health, operating room, nursing education, nursing supervisors, radiology, infection control, nutrition care, case management, information management division, logistics, environmental services, and clinical nurse specialists (CNS) from the center for nursing science and clinical inquiry. The CIWG commenced in early March 2020 and was a crucial first step to help the organization adapt to a changing environment and requirements. The team met daily before the first patient’s arrival to discuss recent developments, requirements, training needs, pending admissions or inbound aeromedical evacuations, and plan patient care. Regular meetings allowed team members to review the latest COVID-19 guidance and adjust plans and procedures based on evolving evidence. The team developed organizational standard operating procedures (SOPs) that guided all COVID-19 activities related to patient care, workflow, and training. The COVID-19 SOP’s purpose was to guide the diagnosis, disposition, and treatment of either COVID-19 positive patients or those suspected of having the disease. The COVID-19 SOP is a living document that is updated regularly to reflect the pandemic’s rapidly changing nature. The SOP outlines the procedures for hospital and inpatient staff to provide optimal patient care while minimizing the risk of transmission. We designed the SOP to serve as a “quick reference” to on-call personnel at the point-of-care. The SOP is not a comprehensive policy and is not a substitute for clinical judgment.

## BED EXPANSION PLAN

Caring for COVID-19 positive and negative patients created workflow, space, and safety problems, as leaders enacted measures to decrease the spread of the disease to uninfected patients. First, nursing leadership and section leaders completed a thorough facility assessment, including inpatient and outpatient areas, verified current capabilities, and identified expansion contingency wards and equipment needs. As a result of the walkthrough, a seven-tiered bed expansion plan containing distinct COVID units was developed to actively manage inpatient beds for positive and suspected COVID-19 patients. The inpatient leadership team developed the bed expansion plan as the way forward after working through multiple courses of actions with the command team. The first unit developed in the tiered expansion plan was an 11-bed COVID-19 Unit (CVU) with critical care capabilities. To ensure adequate critical care support and close proximity to the emergency department, the hospital commander directed the ambulatory procedure unit’s repurposing into the CVU, serving as the central hub for all COVID-19 patient admissions in Tier-1. As the number of patients increased, the next unit’s activation would occur once the current unit reached a 75% occupancy rate. To date, inpatient COVID-19 support operation continues in CVU, and the hospital remains in the Tier-1 of the bed expansion plan.

In preparation for the COVID-19 response and bed expansion plan, the hospital commander reduced outpatient clinical face-to-face appointments by 80%. Thus, to limit the spread of the disease, most patients received healthcare from their providers via a virtual platform. The move to a virtual format mitigated disease transmission risk while still enabling face-to-face appointments for patients requiring high-risk screenings. To date, clinical operations have returned to 60% face-to-face with COVID-19 precautions, with the remainder occurring via the virtual platform.

The department of surgery reduced operations by 80% to strictly emergent and urgent procedures, enabling the conversion of the ambulatory procedure (APU) and same day surgery (SDS) units to the specialized CVU that is still in current operation. The administrative offices, including support for critical care CNS, were also relocated. To date, surgeons are operating at nearly 70% of the pre-COVID-19 capacity, with the SDS and APU continuing to occur in their new locations. The change in the COVID-19 operational tempo allowed the CIWG to reduce team administrative huddles to monthly, with clinical leader consultation as needed. The daily Multi-D clinical rounds continue when there are patients on the CVU.

## WORKFLOW

The CIWG made changes to the workflow for each unit to ensure safety, limit traffic in and out of the unit, and decrease the disease’s spread. The CIWG developed workflow diagrams that established entry and exit points for staffing and patient transport, a donning and doffing area, and a dedicated team locker room and rest area. One elevator was designated as the transport for COVID-19 patient movement. Access points to the unit were restricted to only CVU and CIWG team members to reduce traffic entering the unit. To manage patient flow, the CIWG developed three categories of COVID-19 patient admission criteria: positive, probable, and unlikely. Confirmed symptomatic positive and probable patients were admitted to the CVU. Patients with an atypical clinical presentation were admitted to an isolation or private room in the medical-surgical (MEDSURG) ward, pending COVID-19 test results.

## RESOURCE MANAGEMENT

### Supplies and Equipment

The ongoing wartime mission and readiness posture for contingency operations placed LRMC at an advantage for the COVID-19 response compared with other facilities concerning supply chain management. However, effective collaboration with hospital logistics and facilities was paramount to acquire the equipment (ventilators, monitors, and UV sterilization) and the renovation of ventilation systems necessary to support the mission. The logistics division maintains mass casualty supplies and equipment for contingency operations and collaborates with the regional strategic supply chain to ensure the availability of expansion equipment, including ventilators, monitors, and PPE. On short notice, the team acquired additional transport monitors, beds, and ventilators to nearly double pre-COVID-19 operational bed capacity. To date, the LRMC COVID-19 response has not exceeded the Tier-1 bed capacity, and we have had an excess of hospital beds and ventilators. The one item that we certainly maximized from the contingency stocks was PPEs. A clear reporting process and open lines of communication between unit leaders and logistics ensured that the CVU had the necessary supplies and equipment to provide safe patient care.

### Personal Protective Equipment

**FIGURE 1.**
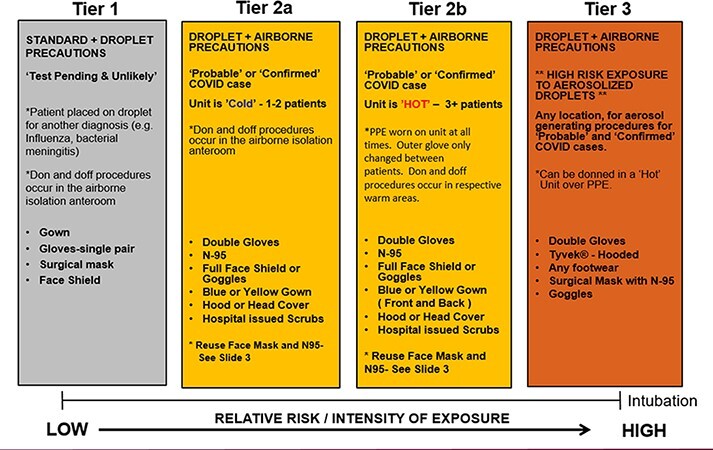
Landstuhl Regional Medical Center personal protective equipment (PPE) guidance.

In the early phases of the pandemic, PPE projection, availability, timely acquisition, and resupply were top priorities. Operational plans guided PPE requirements by estimating the number of personnel per shift, the number of shifts, and work/rest cycle. PPE burn rates depended on multiple variables, including PPE protocols (Fig. 1) and the number of staff-to-patient encounters. We adapted the Centers for Disease Control and Prevention (CDC) PPE burn rate calculator^11^ as a template to provide a foundation for establishing requirements. The original CDC calculator helped track how quickly PPE was being used, rather than PPE demand projections. The CDC calculator was modified to include the staff roles, number of staff per shift, number of shifts, number of patients, and number of potential staff to patient encounters based on patient acuity.

COVID-19 Inpatient Working Group developed this PPE worksheet as a guide for donning and doffing PPEs by nursing personnel. The standard was utilized as a checklist for donning and doffing protective equipment and was strictly enforced.

We validated the PPE calculator after caring for the first COVID patient. The CNS tracked the number of staff to patient encounters, and the number of each PPE items was logged. Hospital leaders used the data for decision support to decide if clinicians should reuse PPE when faced with 10+ patients. The team compared the initial burn rate projection with actual burn rate data for a given patient over 10 days. The amount of PPE used throughout the patient’s stay remained consistent even as the patient converted from an ICU patient to a MEDSURG patient. The type of events that drastically increased the PPE usage were new patient admissions, proning procedures, intubating, and patient extubation. To date, clinical staff members have not reused PPE due to shortages.

## STAFFING PLAN

Early in the operation’s planning phase, clinical leaders determined that staffing was a major limiting factor in the response plan. Nurse staffing for critical care support represented the largest projected manpower deficit. The team calculated the staffing deficit based on the DoD COVID-19 Practice Management Guide (PMG), which estimated that 80% of COVID-19 projected cases would be mild, 15% severely ill, and 5% would require critical care support and interventions, including mechanical ventilation.^12^ Other issues that further complicated staffing challenges were routine patient care operations, including combat casualty care and the level of turbulence associated with the air evacuation and critical care transport mission. The average daily census for routine patients and battle injuries in the ICU and MEDSURG unit varied daily. The patient load from inbound aeromedical flights was unpredictable; however, the number of weekly outbound fights was consistent. To mitigate staffing challenges, the hospital commander temporarily closed clinic operations, enabling clinic nursing personnel to train and support the inpatient mission. As a result, frontline nursing leaders developed a dedicated team of nurses and technicians from the ICU and MEDSURG wards to provide direct patient care in the CVU. The rest of the nursing staff from both units, supported the non-COVID-19 MEDSURG and critical care operations with augmentation from sister services and clinics to include labor and delivery, mother-baby unit, outpatient clinics, and the department of surgery. Nursing leaders developed a team nursing staffing plan based on the PMG crisis staffing model to support direct patient care. A care team included an ICU Registered Nurse (RN), a MEDSURG RN, a Licensed Practical Nurse, and a technician. Initially, another technician was posted at the entrance and exit of the ward to assist and monitor the donning and doffing of PPE, respectively. The CVU implemented a buddy system for donning and doffing of PPE. We grouped care to conserve PPE and reduce the number of times the staff entered the room. As census, acuity and/or workload increased, the team adjusted the staffing plan. This staffing mix allowed care-teams to manage multiple patients with work–rest cycles and ultimately became the standard of care, which continues to be utilized today.

## JUST-IN-TIME TRAINING

**FIGURE 2.**
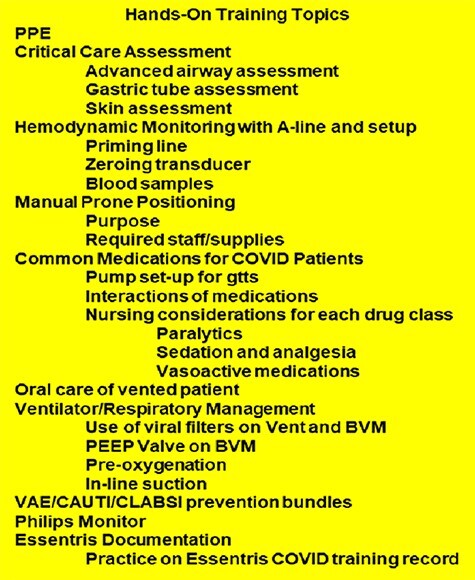
Just-in-time training modules and topics from society of critical care medicine.

The CIWG education team planned and implemented just-in-time training (JTT) for inpatient and outpatient clinical nurses to learn the requisite skills necessary to augment critical care nursing areas. The staff were able to be cross-leveled after receiving the JTT for the personnel assigned to inpatient services, the primary care clinics, and other departments. The education team consisted of senior critical care nurses, respiratory therapy technicians, critical care, and MEDSURG CNSs. The team used online modules from the Society of Critical Care Medicine ^13^ to provide non-ICU clinicians with baseline education on COVID-19 critical care treatment. The team also provided a 2-hour simulation-based training session in the ICU with a high-fidelity mannequin. The topics covered in the hands-on training were crucial elements of ICU care, including ventilator, hemodynamics, telemetry basics, and proning procedures (Fig. 2). In addition to the learning modules and hands-on ICU training, the education team provided PPE training for nursing personnel to prevent cross-contamination and potential exposure to the disease. Last, the non-critical care nurses went through a short orientation and preceptorship with an ICU nurse and fellow teammates already working on the COVID-19 units.

Clinical staff were assigned modules online to complete as a prerequisite. They would then schedule to attend a simulation-based familiarization training with basic ICU equipment and patient management skills. As stated above the training team made sure to address topics treatment protocols that were related to managing a COVID-19 patient.

## COMMUNICATION

### Team Huddles

The CIWG met daily to plan operational activities, discuss real-time issues and challenges, and develop real-time Multi-D solutions. Starting in the early phase of COVID-19, the CIWG meetings were held in-person with social distancing and mask, and then virtually. The meeting promoted robust discussions, brainstorming of ideas, and open expression of views without fear of reprisal or discipline. The CIWG continually adapted patient care, processes, and safety measures to new guidance from the DoD, CDC, LRMC infectious diseases, and current clinical activities. The forum allowed for decision-making at the tactical level. However, senior hospital leadership made final decisions on operational plans that impacted the larger organizational mission. A decision to create a COVID-19 specific code team using Vocera, a handheld communication system staff wore on their uniform. This method of communication ensured an efficient and safe emergency response to a COVID-19 patient in crisis.

### Multidisciplinary Rounds

The CIWG conducted daily Multi-D rounds in the CVU. The purpose of the dedicated rounds was to discuss the status of each COVID-19 patient, care plan, and care coordination issues, and, to provide any updates on guidelines or standards to the team. The team also discussed projected inbound patients to LRMC from deployed or remote areas in the LRMC area of responsibility, including Europe, Southeast Asia, and Africa. The team conserved inpatient beds, supplies, and resources by isolating the COVID-19 patients coming from downrange that did not meet the criteria for hospitalization in isolation lodging on the LRMC instillation. Key factors that enabled positive patient outcomes in the COVID-19 ICU were nutrition care and early rehabilitation. Physical therapists (PT) and PT technicians often performed therapy, such as walking with critically ill patients, inside the hospital to facilitate recovery. The team recognized this could increase the potential for exposure to the disease between patients and staff. To maintain standard of care and optimize recovery for this population, PT conducted exercise outside while following established PPE protocols. The nutrition care division delivered meals at the CVU entrance in disposable containers.

### Unit-Level Communication with Frontline Staff

Leadership engagement and open lines of communication between LRMC leaders and frontline staff promoted a healthy work environment and enabled safe and efficient clinical operations. Unit-level leaders conducted daily staff safety huddles to provide assurances about operational safety, encouraged self-care, and provide resources. One of the first things that instilled a sense of ownership and support from the command team was when the Chief Nursing Officer provided direct patient care to the first intubated critically ill COVID-19 patient. This action communicated to the unit leaders and the staff that the hospital leaders would not put their health at risk. Unit leaders paid special attention to signs of complacency with safety protocols and any mental health issues related to feelings of isolation from other team members not involved in caring for COVID-19 patients. Early intervention through leadership engagement, resource management, training, and leveraging existing programs such as pastoral care and clinical psychology to support self-care were critical for staff resilience.

## LESSONS LEARNED

The LRMC clinical leader initiative to develop the CIWG led to a coordinated pandemic response while maintaining routine combat casualty care support. In developing and executing a response plan, some key lessons learned centered on teamwork, communication, adapting to change, and leadership.

### Teamwork

Key to the success of CIWG was an intentional effort in the early phase of the pandemic to bring a Multi-D team together to work toward a common goal through knowledge sharing and collaboration. In so doing, we executed a coordinated response to the pandemic, members of the team learned capabilities and limitations of the facility, relationships were built, partnerships were forged, and issues were addressed in a timely manner.

### Communication

Inpatient operations were heavily staffed by a novice nursing team, JTT, and staying vigilant with closed-loop communication within the team and frontline staff was important for success. In addition to communicating new information to the staff during team huddles, leaders helped subordinates understand the rationale behind changes and new guidance. Face to Face (F2F) or Virtual Telehealth (VTH) and Multi-D rounds kept everyone on the same page, and issues were addressed early. Open lines of communication with the hospital commander through the Deputy Commander for Inpatient Services level of leadership created a quicker channel for making decisions that impacted the organization. Also vital to operation were open lines of communication with nursing supervision. We noted confusions between clinical staff and nursing supervisors in the early phases of inpatient care that were not in sync. The fact that this team did not play a very active role in the planning phase for the pandemic response resulted in confusion with bed management and compliance with established admission processes. Thus, these issues had to be resolved in the middle of a real pandemic response.

### Adapting to Change

Quickly adapting to an evolving situation was important for the success of the response plan and the team. This operation evolved overtime with changing information, practices, and guidelines from multiple avenues to include the CDC and World Health Organization. The policies and plans developed were kept fluid and updated almost daily. The updated policies were stored on the hospital intranet, enabling easy access by leaders and clinicians facility-wide. Within the CIWG, LRMC had a core group of expert decision-makers who oversaw the ground-level response and quickly adapted policies and staffing to meet standards.

### Leadership

Inpatient services also succeeded in responding to the pandemic because we largely abandoned the more rigid leadership framework and traditional approval process for decision-making in exchange for speed. CIWG leaders were empowered to make decisions using the best evidence available without lengthy bureaucratic approval processes. The team had open lines of communication with senior leaders, and they were engaged as needed. Department chiefs empowered their junior leaders and staff to be creative and offer novel solutions to address identified problems. Leaders assigned tasks to team members regardless of rank because they showed initiative and follow-through.

## CONCLUSIONS

The LRMC mission statement is: “LRMC, with partners, elevates the readiness and healthcare support of our joint warfighters and their families, by maximizing the quality and safety of our Role-4 Theater Hospital across the continuum of care in support of the Combatant Commanders.” The assumption as an MTF is that the hospital and team are trained and ready to respond to this call and to any crisis, if and when overwhelmed, the host nation will be able to support. This pandemic and its impact on healthcare systems across the globe challenged these assumptions. The constant preparation and training for contingency operations and, most importantly, the development of the CIWG working toward a common goal led to a successful LRMC readiness and response to the COVID-19 pandemic while continuing to support the joint warfighter. The CIWG was not only able to develop and execute an inpatient response to the pandemic, but it was also able to assess, analyze, and adapt to internal challenges and new guidelines along the way. Future and current leaders can use our implementation strategies and lessons learned to prepare for the next pandemic response. Many lessons were learned in teamwork, communication, adapting to change, and leadership applied in real time to operation with quality and safety as our center of gravity.
